# Prediction Models and Their External Validation Studies for Mortality of Patients with Acute Kidney Injury: A Systematic Review

**DOI:** 10.1371/journal.pone.0169341

**Published:** 2017-01-05

**Authors:** Tetsu Ohnuma, Shigehiko Uchino

**Affiliations:** 1 Intensive Care Unit, Department of Anesthesiology, Saitama Medical Center, Jichi Medical University, Saitama, Japan; 2 Intensive Care Unit, Department of Anesthesiology, Jikei University School of Medicine, Tokyo, Japan; University of Sao Paulo Medical School, BRAZIL

## Abstract

**Objectives:**

To systematically review AKI outcome prediction models and their external validation studies, to describe the discrepancy of reported accuracy between the results of internal and external validations, and to identify variables frequently included in the prediction models.

**Methods:**

We searched the MEDLINE and Web of Science electronic databases (until January 2016). Studies were eligible if they derived a model to predict mortality of AKI patients or externally validated at least one of the prediction models, and presented area under the receiver-operator characteristic curves (AUROC) to assess model discrimination. Studies were excluded if they described only results of logistic regression without reporting a scoring system, or if a prediction model was generated from a specific cohort.

**Results:**

A total of 2204 potentially relevant articles were found and screened, of which 12 articles reporting original prediction models for hospital mortality in AKI patients and nine articles assessing external validation were selected. Among the 21 studies for AKI prediction models and their external validation, 12 were single-center (57%), and only three included more than 1,000 patients (14%). The definition of AKI was not uniform and none used recently published consensus criteria for AKI. Although good performance was reported in their internal validation, most of the prediction models had poor discrimination with an AUROC below 0.7 in the external validation studies. There were 10 common non-renal variables that were reported in more than three prediction models: mechanical ventilation, age, gender, hypotension, liver failure, oliguria, sepsis/septic shock, low albumin, consciousness and low platelet count.

**Conclusions:**

Information in this systematic review should be useful for future prediction model derivation by providing potential candidate predictors, and for future external validation by listing up the published prediction models.

## Introduction

Acute kidney injury (AKI) is a common complication among critically ill patients and their mortality is high [1–4]. Reliable AKI specific scoring systems are important to predict outcome of AKI patients and to provide severity stratification for clinical studies. However, general severity scores for critically ill patients, e.g., Acute Physiology and Chronic Health Evaluation (APACHE) [5–7], Simplified Acute Physiology Score (SAPS) [8, 9], and Mortality Probability Model [10] have shown controversial results on the accuracy of predicting mortality in AKI patients [11–13], partly because those scores were generated from data that included only a few AKI patients.

Over the past three decades, multiple AKI outcome prediction models, which incorporated physiologic, laboratory, organ dysfunction and previous comorbidity, have been derived [14–20]. Even in the 21^st^ century, five additional prediction models have been generated [12, 21–24]. Although internal validation of these prediction models has shown good accuracy, the results of external validation studies for the models have been unsatisfactory [11, 25, 26]. Currently, there is neither consensus nor guideline recommending which prediction model to apply to clinical practice.

The objectives of this study are to systematically review the AKI outcome prediction models and their external validation studies, to describe the discrepancy of reported accuracy between the results of internal and external validations, and to identify variables frequently included in the prediction models, which might be potentially useful for future prediction model derivation.

## Materials and Methods

### Studies eligible for review

Studies published in the medical literature were eligible if they derived a model to predict mortality of AKI patients or externally validated at least one of the prediction models, and presented area under the receiver-operator characteristic curves (AUROC) [27] or the concordance index (c-statistic) to assess model discrimination. Studies were excluded if they described only results of logistic regression without reporting a scoring system, or if a prediction model was generated from a specific cohort. Unpublished conference abstracts were also excluded. This study followed the same principal as in the Preferred Reporting Items for Systematic Reviews and Meta-Analyses (PRISMA) statement (S1 PRISMA Checklist) [28].

### Literature review and study selection

We searched the MEDLINE and Web of Science electronic databases (until January 2016). In the MEDLINE search, we used the terms of “acute kidney injury” (MeSH Terms), “statistical model” (MeSH Terms), “predictive value of tests” (MeSH Terms) and “validation”. In the Web of Science, we used Key words of “acute kidney injury”, “acute renal failure”, “model”, “prediction”, “predictor”, “validity”, and “validation”. References of all selected articles were searched to identify any eligible studies. The search was restricted to human subjects. Each article selected by the primary reviewer (TO) was assessed by the second reviewer to confirm eligibility (SU).

### Data extraction

A standardized data abstraction form was used to collect data on study characteristics and outcomes of interest. Data collected to describe characteristics of articles for original outcome prediction models were the type of study, study period, number of centers, sample size, mean age, gender, region, population, renal replacement therapy (RRT) requirement, hospital mortality, AKI definition, exclusion criteria, follow-up and variables included in prediction models. Following information was also collected for quality assessment of the prediction models: definition of predictors, indications for RRT defined, missing data definition, bootstrap resampling, multivariable analysis approach, event per variable ratio and internal validation cohort.

Data collected to describe characteristics of articles for external validation were type of study, study period, number of centers, sample size, mean age, hospital mortality, number of validated models and methods of discrimination and calibration. AUROCs reported in both original prediction models and external validation studies were also collected.

## Results

**Fig 1:**
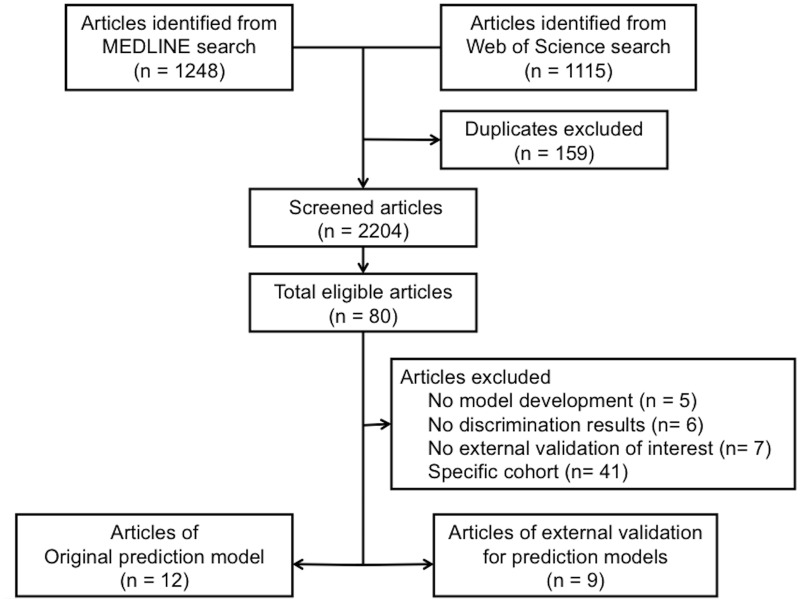
Selection of articles by PRISMA flow diagram

A total of 2204 potentially relevant articles were found and screened, of which 80 were retrieved for detailed evaluation (Fig 1). We excluded five articles that had no prediction models developed by multivariate regression analysis, six articles that had no discrimination results, seven articles that validated only general severity scores or had no external discrimination results and 41 articles that assessed specific cohorts (cardiac surgery: 10, contrast-induced nephropathy: eight, others: 23). Fifty-nine articles excluded from this study are listed in a supplement file (S1 File). Finally, 12 articles reporting original prediction models for hospital mortality in AKI patients [12, 14–24] and nine additional articles assessing external validation of the outcome prediction models [11, 25, 26, 29–34] were selected for analysis. Five out of 12 articles reporting original prediction models also assessed other models (14 articles in total for external validation).

Characteristics of the 12 articles reporting outcome prediction models for AKI are shown in Tables 1 and 2. The study sample size ranged from 126 to 1,122 patients and the hospital mortality ranged from 36% to 75%. Only five studies (Chertow 1998, Mehta, Lins 2004, Chertow 2006, Demirjian) included more than one center and remaining seven were conducted in single center. The definition of AKI was not uniform among the 12 articles and none used recently published consensus definitions for AKI. Quality assessment for these articles is shown in Table 3. How missing data were dealt was defined only in four articles, and all of these articles also used bootstrap resampling. Eight articles used multivariable logistic regression analysis, and the other four articles (Ramussen, Schaefer, Liano and Lins 2000) used multivariable linear regression analysis. The event per variable ratio was more than 10 in all articles except for the earliest (Ramussen).

**Table 1:** Characteristics of articles reporting outcome prediction models for acute kidney injury.

	Type of study	Study period	Centers, Number	Sample size	Mean age, years	Gender, Male %	Region	Population	RRT requirement	Hospital mortality
Ramussen 1985 [14]	Retrospective	1977–1981	1	148	58	NR	Australia	Hospital	50%	53%
Lohr 1988 [15]	Retrospective	1979–1985	1	126	57	73%	USA	Hospital	100%	75%
Schaefer 1991 [16]	Prospective	1985–1988	1	134	NR	NR	Germany	ICU	100%	57%
Liano 1993 [17]	Prospective	1977–1988	1	328	57	81%	Spain	Hospital	51%	53%
Paganini 1996 [18]	Retrospective	1988–1992	1	506	63	61%	USA	ICU	100%	67%
Chertow 1998 [19]	Post hoc of RCT	1993–1995	59	256	62	65%	USA, Canada	Hospital	42%	36%
Lins 2000 [20]	Prospective	1996–1997	1	197	70	60%	Belgium	ICU	26%	53%
Mehta 2002 [12]	Prospective	1989–1995	4	605	56	72%	USA	ICU	50%	52%
Lins 2004 [21]	Prospective	1997–1998	8	293	72	62%	Belgium	ICU	37%	51%
Dharan 2005 [22]	Prospective	2002	1	265	48	71%	India	Hospital	26%	38%
Chertow 2006 [23]	Prospective	1999–2001	5	618	59	59%	USA	ICU	64%	37%
Demirjian 2011 [24]	Post hoc of RCT	2003–2007	27	1,122	60	71%	USA	ICU	99%	50%

RRT: renal replacement therapy, NR: not reported, RCT: randomized control trial.

**Table 2:** AKI definitions, exclusion criteria and follow-up of articles reporting outcome prediction models for acute kidney injury.

	AKI definitions	Exclusion criteria	Follow-up
Ramussen 1985 [14]	SCR > 2.0 mg/dl, or more than 50% elevation if baseline SCR > 1.7mg/dl,	Glomerulonephritis, uric acid nephropathy, ureteric obstruction, interstitial nephritis	Hospital
Lohr 1988 [15]	Requiring RRT	Post-renal transplantation	NR
Schaefer 1991 [16]	Requiring RRT	Chronic HD and kidney transplantation	ICU
Liano 1993 [17]	SCR >2.0 mg/dl	Previous renal failure and hepato-renal syndrome, vascular, interstitial, glomerular, obstructive etiology	NR
Paganini 1996 [18]	Requiring RRT	Less than 18 years	NR
Chertow 1998 [19]	SCI of >1mg/dl	Pre-renal azotemia, urinary obstruction, glomerulonephritis, interstitial nephritis, CKD, renal transplantation	30-day
Lins 2000 [20]	SCR > 2.0 mg/dl or more than 50% elevation		Hospital
Mehta 2002 [12]	SCR ≥ 2.0 mg/dl, BUN ≥ 40 mg/dl, or SCI ≥ 1.0 mg/dl with preexisting renal insufficiency	Previous dialysis, kidney transplantation, urinary obstruction, hypovolemia	Hospital
Lins 2004 [21]	SCR >2.0 mg/dl or >50% increase in preexisting mild to moderate renal disease	Baseline SCR >3.0 mg/dl	Hospital
Dharan 2005 [22]	SCI ≥ 0.5 mg/dl with baseline SCR less than 1.9 mg/dl, or SCI ≥ 1.0 mg/dl with baseline SCR between 2.0 to 4.9 mg/dl	Baseline SCR >5.0 mg/dl, transplant recipients	NR
Chertow 2006 [23]	SCI ≥ 0.5 mg/dl with baseline SCR < 1.5 mg/dl, or SCI ≥ 1.0 mg/dl with baseline ≥ 1.5 mg/dl and < 5.0 mg/dl	Baseline SCR ≥ 5.0 mg/dl, previous HD, kidney transplantation, urinary tract obstruction	Hospital
Demirjian 2011 [24]	Ischemic or nephrotoxic ATN, oliguria, SCR ≥ 2 mg/dl in males or ≥ 1.5 mg/dl in females	Baseline SCR > 2.0 mg/dl in males, > 1.5 mg/dl in females, previous HD, kidney transplant	60-day

AKI: acute kidney injury, RRT: renal replacement therapy, SCR: serum creatinine, SCI: serum creatinine increase, ATN: acute tubular necrosis, CKD: chronic kidney disease, HD: hemodialysis, NR, not reported.

**Table 3:** Quality assessment for articles reporting outcome prediction models for acute kidney injury.

	Definition of predictors	Indications for RRT defined	Missing data definition	Bootstrap resampling	Multivariable analysis approach	Event per variable ratio	Internal validation cohort
Ramussen 1985 [14]	Yes	Yes	NR	NR	Stepwise multiple linear regression	7.9 (10/79)	Split sample
Lohr 1988 [15]	Yes	Yes	NR	NR	Stepwise logistic regression	31.3 (3/94)	NR
Schaefer 1991 [16]	Yes	Yes	NR	NR	Stepwise linear discriminant procedure	12.7 (6/76)	NR
Liano 1993 [17]	Yes	Yes	NR	NR	Multiple linear regression	19.3 (9/174)	Cross-validation
Paganini 1996 [18]	Yes	NR	NR	NR	Stepwise logistic regression	43.0 (8/344)	Both
Chertow 1998 [19]	NR	Yes	Yes	Yes	Logistic regression	10.6 (7/74)	NR
Lins 2000 [20]	Yes	NR	NR	NR	Linear regression	20.8 (5/104)	Split sample
Mehta 2002 [12]	Yes	NR	Yes	Yes	Stepwise backward logistic regression	34.9 (9/314)	Split sample
Lins 2004 [21]	Yes	NR	NR	NR	Logistic regression	18.3 (8/146)	Split sample
Dharan 2005 [22]	Yes	NR	NR	Yes	Logistic regression	10.0 (10/100)	Both
Chertow 2006 [23]	Yes	Yes	Yes	NR	Stepwise backward logistic regression	32.7 (7/229)	NR
Demirjian 2011 [24]	Yes	Yes	Yes	Yes	Stepwise backward logistic regression	28.3 (21/595)	Split sample

NR: not recorded.

Characteristics of the 14 external validation studies are shown in Table 4. The study sample size ranged from 197 to 17,326 patients and the hospital mortality ranged from 37% to 85%. Five studies were conducted in single center. All studies evaluated discrimination with the AUROC and nine studies evaluated calibration with the Hosmer-Lemeshow test.

**Table 4:** Characteristics of external validation studies for acute kidney injury outcome prediction models.

	Type of study	Study period	Centers, Number	Sample size	Mean age, years	Hospital mortality	Validated models	Discrimination	Calibration
Douma 1997 [11]	Retrospective	1985–1993	1	238	61	76%	4	AUROC	H-L
Lins 2002 [21]	Prospective	1996–1997	1	197	70	53%	1	AUROC	NR
Martin 2002 [29]	Retrospective	1995–1996	1	349	58	59%	2	AUROC	NR
Mehta 2002 [12]	Prospective	1989–1995	4	605	56	52%	7	AUROC	H-L
d’Avila DO, 2004 [30]	Prospective	NR	1	280	54	85%	1	AUROC	H-L
Dharan 2005 [22]	Prospective	2002	1	265	48	38%	1	AUROC	H-L
Uchino 2005 [26]	Prospective	2000–2001	54	1,742	67	61%	4	AUROC	H-L
Lima 2005 [31]	Prospective	2000–2001	1	342	60	85%	1	AUROC	H-L
Chertow 2006 [23]	Prospective	1999–2001	5	618	59	37%	4	AUROC	NR
Kolhe 2008 [25]	Retrospective	1995–2004	170	17,326	63	59%	3	AUROC	H-L
Lin 2008 [32]	Retrospective	2002–2005	4	334	64	66%	4	AUROC	NR
Costa e Silva VT 2009 [33]	Prospective	2003–2005	1	366	NR	68%	3	AUROC	H-L
Demirjian 2011 [24]	Post hoc of RCT	2003–2007	27	1,122	60	50%	1	AUROC	NR
Ohnuma 2015 [34]	Retrospective	2010	14	343	69	59%	6	AUROC	H-L

RCT: randomized controlled trial, NR: not recorded, AUROC: area under the receiver operating characteristic curve, H-L: Hosmer-Lemeshow.

**Fig 2:**
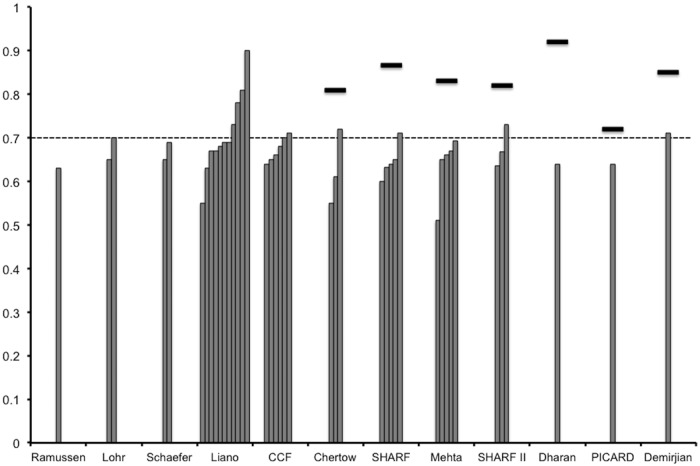
Area under the receiver operating characteristic curves (AUROC) for hospital mortality reported in the original articles and external validation studies. Black horizontal bars: AUROC in original studies, gray columns: AUROC in external validation studies.

AUROCs for hospital mortality reported in the original articles (internal validation) and external validation studies are shown in Fig 2. Seven recently published articles for AKI outcome prediction models reported AUROCs for internal validation and all of them had high AUROCs of above 0.7. All prediction models were externally validated by one or more studies. AUROCs in the external validation studies for these scores were generally low (less than 0.7 in most studies). In addition, seven prediction models that were validated both internally and externally had invariably lower AUROCs in external validation than those in internal validation.

Table 5 shows variables included in more than one prediction model and their odds ratios / p values. There were 10 common non-renal variables that were reported in more than three prediction models: mechanical ventilation, age, gender, hypotension, liver failure, oliguria, sepsis/septic shock, low albumin, consciousness and low platelet count. Renal variables (low creatinine and high urea) were often used in the same prediction models.

**Table 5:** Variables included in more than one prediction model and their odds ratios / p values.

	Number of studies	Ramussen	Lohr	Schaefer	Liano	Paganini	Chertow1998	Lins2000	Mehta	Lins2004	Dharan	Chertow2006	Demirjian
Mechanical ventilation	9	-	NR	P<0.01	P<0.01	NR	2.95	10.2	-	NR	2.3	-	P<0.01
Age	7	-	-	-	P<0.01	-	-	1.70	1.02	NR	1.0	1.16	P<0.01
Gender	5	-	-	-	P<0.05	NR	3.70	-	2.36	-	0.6	-	-
Hypotension	5	-	NR	P<0.01	P<0.05	-	-	-	-	NR	3.1	-	-
Liver failure	5	-	-	-	P<0.05	NR	-	-	3.06	-	3.7	1.76	-
Oliguria	5	NR	-	-	P<0.05	-	4.39	-	-	-	4.9	-	P = 0.02
Sepsis/septic shock	5	-	NR	NS	-	-	-	-	-	NR	2.2	1.69	-
Low albumin	4	-	-	-	-	-	-	1.50	-	NR	1.7	-	P = 0.02
Consciousness	4	NR	-	-	P<0.05	-	7.35	-	-	-	10.4	-	-
Low platelet count	4	-	-	-	-	NR	-	-	3.40	-	-	2.10	P<0.01
Heart failure	3	-	NR	-	-	-	-	1.88	-	NR	-	-	-
Preexisting heart disease	3	NR	-	NS	-	-	-	-	-	-	-	-	P = 0.03
Prothrombin time	3	-	-	-	-	-	-	1.29	-	NR	-	-	P = 0.01
Respiratory failure	3	NR	-	-	-	-	-	-	2.62	-	-	1.84	-
Surgical patients	3	NR	-	-	-	NR	-	-	-	-	-	-	P = 0.08
High bilirubin	2	-	-	-	-	-	-	-	-	NR	-	-	P<0.01
Acute cardiac illness	2	NR	-	-	-	-	5.90	-	-	-	-	-	-
Bicarbonate	2	-	-	-	-	-	0.93	-	-	-	-	-	P = 0.02
Malignancy	2	NR	-	-	-	-	-	-	-	-	-	-	P<0.01
Low creatinine	4	-	-	-	-	NR	-	-	0.71	-	-	0.81	P = 0.01
High urea	3	-	-	-	-	NR	-	-	1.02	-	-	1.09	-

NR: not reported, NS: not significant.

## Discussion

### Key findings

We have systematically reviewed AKI outcome prediction models and their external validation studies. We found 12 articles reporting original prediction models for hospital mortality in AKI patients and nine articles assessing external validation of the outcome prediction models. Although good performance was reported in their internal validation, most of the prediction models had poor discrimination with an AUROC below the threshold of 0.7 in their external validation studies. We also identified 10 common variables that were frequently included in the prediction models.

### Relationship to previous studies

The establishment of a clinical prediction model encompasses three consecutive research phases, namely derivation, external validation and impact analysis [35]. In this study, we conducted a systematic review for the first two phases in AKI outcome prediction. Several systematic reviews for clinical prediction models and their external validation have been conducted in other medical conditions, which consistently found methodological limitations. [36–40]. Such limitations include case mix heterogeneity, small sample sizes, insufficient description of study design, and lack of external validation. We found the same limitations in the AKI outcome prediction studies. For example, all prediction models examined in this study were relatively old (data collected more than 10 years ago) and conducted before consensus criteria for AKI were published [41–43]. Therefore, patients included in these prediction models were heterogeneous, with varied RRT requirement and mortality. We also found that more than half of the studies for AKI prediction models and their external validation were single-center (12/21, 57%), and most of them included less than 1,000 patients (19/22, 86%). Furthermore, the moment of data collection for each clinical prediction model and external validation was different. Data collection can be done at admission, at AKI diagnosis, at the start of RRT, at nephrologist consultation, and so on. Demirjian’s model for instance, collected variables at RRT start [24], while other models collected variables at nephrologist consultation [12, 23], or at AKI diagnosis [21, 23]. This variable is also important for external validation, as the discrimination AUROC value can be altered if variables are collected at different moments in the new cohort. Considering the poor generalizability of currently available prediction models (AUROCs lower than 0.7 in most external validation studies), a large database collected in multicenter using consensus AKI criteria will be needed both to derive and validate AKI outcome prediction models.

Among the prediction models included in this systematic review, we found that the Liano’s score [17] was the most often evaluated externally (11 studies). The range of AUROC validated externally for the Liano’s score was from 0.55 to 0.90, and four of them were above 0.7. The reason why Liano showed high AUROCs in some external validation studies is unclear. It might be partially explained by that the Liano’ score contained several risk factors that are frequently used in the prediction models (mechanical ventilation, age, gender, hypotension, liver failure, oliguria, consciousness disturbance), although Dharan also included nine variables, with poor discrimination by one external validation study (Table 5).

### Significance and implications

To derive an accurate prediction model, choosing appropriate candidate predictors is of much importance. Previous studies have shown that clinical intuition may not be suitable for identifying candidate predictors [44]. A better approach is to combine a systematic literature review of prognostic factors associated with the outcome of interest with opinions of field experts [35]. We identified 10 common variables that were frequently included in the prediction models. These variables are also often found to be related to mortality in more recent epidemiological studies using consensus AKI criteria [45–48]. We believe that our study results will be useful for future studies to derive accurate AKI outcome prediction models by including these variables for data collection.

Although often included in the prediction models, we think that including both low creatinine and high urea concentrations as independent variables can be problematic (Table 5). Low serum creatinine is included in general severity scores as one of independent variables [5]. Serum urea has been used as a marker of timing of starting RRT in several studies, which showed that patients with higher urea at start of RRT had worse outcome than patients with lower urea [49]. High urea is also included in general severity scores [5]. However, serum creatinine and urea concentrations clearly have strong co-linearity. In AKI patients, urea is almost always high when creatinine is high. Even if both variables are found to be independent variables in multivariable analysis, it seems unlikely that including both variables in a prediction model will improve prediction ability [50].

Physicians are faced with the impractical situation of having to choose among many concurrent outcome prediction models for AKI. To overcome this issue, it is recommended that investigators who have large data sets should conduct external validation studies of multiple existing models at once, in order to determine which model is most useful [51]. We believe that our study results will also be useful for future studies by providing the list of published outcome prediction models for AKI.

### Strengths and limitations

The strength of our study is that, to the best of our knowledge, this is the first systematic review on AKI outcome prediction models in the medical literature. We have reviewed studies for both prediction models and their external validation, and provided potential candidate variables for future prediction models and the list of published prediction models for future external validation studies.

However, our study also contains several limitations. First, recent studies suggest that AKI biomarkers might be useful to predict outcome and could be combined with physiological and laboratory variables to improve predicting ability [52, 53]. However, prediction models should include only variables that are available at the time when the model is intended to be used, and biomarkers are not yet widely used clinically [54]. Second, we excluded six studies due to discrimination results not available [55–60]. However, these studies were generally old, small, and of poor methodological quality. We believe that including these studies would not change our main findings. Finally, the AKI definitions used in both prediction models and their external validation studies are outdated, and studies included were relatively old (the most recently published study is from 2011 and the data were collected between 2003 and 2007). There is an urgent need for a mortality prediction model based on current definitions of AKI, and this systematic review can be considered a first step to accomplish this task.

## Conclusions

Multiple outcome prediction models for AKI have been derived previously. These scores had good performance in their internal validation studies, while poor performance was reported in their external validation, suggesting that there is no accurate model currently available. To generate accurate AKI prediction models, several recommendations can be provided: using a large database collected in multicenter, applying consensus AKI criteria, and collecting variables frequently used in previous models (mechanical ventilation, age, gender, hypotension, liver failure, oliguria, sepsis/septic shock, low albumin, consciousness and low platelet count). Information in this systematic review should be useful both for future prediction model derivation by providing potential candidate predictors, and for future external validation by listing up the published prediction models.

## Supporting Information

S1 PRISMA Checklist (DOCX)

S1 File Excluded Articles. (DOCX)
