# Supplementary material for: Prediction Models and Their External Validation Studies for Mortality of Patients with Acute Kidney Injury: A Systematic Review
Source: PLoS One. 2017 Jan 5;12(1):e0169341. doi: 10.1371/journal.pone.0169341 (PMC5215838; doi:10.1371/journal.pone.0169341)
Supplement: S1 File — (DOCX) [file pone.0169341.s002.docx]

Articles excluded from the study

1. No model development: [1-5]
2. No discrimination results: [6-11]
3. No external validation of interest: [12-18]
4. Specific cohorts
   - Cardiac surgery: [19-28]
   - Contrast-induced nephropathy: [29-36]
   - Others: [37-59]

**References**

1. Bullock ML, Umen AJ, Finkelstein M*, et al.* The assessment of risk factors in 462 patients with acute renal failure. Am J Kidney Dis 1985; 5: 97-103
2. Garcia-Fernandez N, Lavilla FJ, Rocha E*, et al.* Assessment of haemostatic risk factors in patients with acute renal failure associated with severe systemic inflammatory response syndrome. Development of a prognostic index. Nephron 2002; 92: 97-104
3. Ersoy A, Yavuz M, Usta M*, et al.* Survival analysis of the factors affecting in mortality in injured patients requiring dialysis due to acute renal failure during the Marmara earthquake: survivors vs non-survivors. Clinical Nephrol 2003; 59: 334-40
4. Barrantes F, Feng Y, Ivanov O*, et al.* Acute kidney injury predicts outcomes of non-critically ill patients. Mayo Clin Proc 2009; 84: 410-6
5. Franzen D, Rupprecht C, Hauri D*, et al.* Predicting outcomes in critically ill patients with acute kidney injury undergoing intermittent hemodialysis--a retrospective cohort analysis. Int J Artif Organs 2010; 33: 15-21
6. Cioffi WG, Ashikaga T, Gamelli RL. Probability of surviving postoperative acute renal failure. Development of a prognostic index. Ann Surg 1984; 200: 205-11
7. Lien J, Chan V. Risk factors influencing survival in acute renal failure treated by hemodialysis. Arch Intern Med 1985; 145: 2067-9
8. Corwin HL, Teplick RS, Schreiber MJ*, et al.* Prediction of outcome in acute renal failure. Am J Nephrol 1987; 7: 8-12
9. Barton IK, Hilton PJ, Taub NA*, et al.* Acute renal failure treated by haemofiltration: factors affecting outcome. Q J Med 1993; 86: 81-90
10. Chertow GM, Christiansen CL, Cleary PD*, et al.* Prognostic stratification in critically ill patients with acute renal failure requiring dialysis. Arch Intern Med 1995; 155: 1505-11
11. Radovic M, Ostric V, Djukanovic L. Validity of prediction scores in acute renal failure due to polytrauma. Ren Fail 1996; 18: 615-20
12. Batista PB, Cendorogolo Neto M, dos Santos OF*, et al.* Evaluation of prognostic indexes in critical acute renal failure patients. Ren Fail 2004; 26: 545-52
13. Varricatt VP, Rau NR, Attur RP*, et al.* Validation of Liano score in acute renal failure: a prospective study in Indian patients. Clin Exp Nephrol 2009; 13: 33-7
14. Maccariello E, Valente C, Nogueira L*, et al.* SAPS 3 scores at the start of renal replacement therapy predict mortality in critically ill patients with acute kidney injury. Kidney Int 2010; 77: 51-6
15. Chen J-C, Wang W-J, Wo V-C*, et al.* The ICNARC model is predictive of hospital mortality in critically ill patients supported by acute dialysis. Clin Nephrol 2012; 77: 392-99
16. Halstenberg WK, Goormastic M, Paganini EP. Validity of four models for predicting outcome in critically ill acute renal failure patients. Clin Nephrol 1997; 47: 81-6
17. Ahlstrom A, Kuitunen A, Peltonen S*, et al.* Comparison of 2 acute renal failure severity scores to general scoring systems in the critically ill. Am J Kidney Dis 2006; 48: 262-8
18. Costa e Silva VT, de Castro I, Liano F*, et al.* Performance of the third-generation models of severity scoring systems (APACHE IV, SAPS 3 and MPM-III) in acute kidney injury critically ill patients. Nephrol Dial Transplant 2011; 26: 3894-901
19. Lange HW, Aeppli DM, Brown DC. Survival of patients with acute renal failure requiring dialysis after open heart surgery: early prognostic indicators. Am Heart J 1987; 113: 1138-43
20. Demirjian S, Schold JD, Navia J*, et al.* Predictive models for acute kidney injury following cardiac surgery. Am J Kidney Dis 2012; 59: 382-9
21. Heise D, Kunze N, Buerger J*, et al.* Modifying a kidney injury score by including perioperative data Comparison of three predictive scores. Cent Eur J Med 2012; 7: 420-8
22. Berg KS, Stenseth R, Wahba A*, et al.* How can we best predict acute kidney injury following cardiac surgery?: a prospective observational study. Eur J Anaesthesiol 2013; 30: 704-12
23. Kiers HD, van den Boogaard M, Schoenmakers MCJ*, et al.* Comparison and clinical suitability of eight prediction models for cardiac surgery-related acute kidney injury. Nephrol Dial Transplant 2013; 28: 345-51
24. Kim WH, Lee SM, Choi JW*, et al.* Simplified clinical risk score to predict acute kidney injury after aortic surgery. J Cardiothorac and Vasc Anes 2013; 27: 1158-66
25. Malov AA, Borisov AS, Lomivorotov VV*, et al.* Mortality prediction in patients with dialysis-dependent acute kidney injury after cardiac surgery with cardiopulmonary bypass. Heart lung Circ 2014; 23: 325-31
26. Ng SY, Sanagou M, Wolfe R*, et al.* Prediction of acute kidney injury within 30 days of cardiac surgery. Journal Thorac Cardiovas Surg 2014; 147: 1875-83
27. Kristovic D, Horvatic I, Husedzinovic I*, et al.* Cardiac surgery-associated acute kidney injury: risk factors analysis and comparison of prediction models. Interact Cardiovasc Thorac Surg 2015; 21: 366-73
28. Jorge-Monjas P, Bustamante-Munguira J, Lorenzo M*, et al.* Predicting cardiac surgery-associated acute kidney injury: The CRATE score. J Crit Care 2016; 31: 130-8
29. Chong E, Shen L, Poh KK*, et al.* Risk scoring system for prediction of contrast-induced nephropathy in patients with pre-existing renal impairment undergoing percutaneous coronary intervention. Singapore Med J 2012; 53: 164-9
30. Gurm HS, Seth M, Kooiman J*, et al.* A Novel Tool for Reliable and Accurate Prediction of Renal Complications in Patients Undergoing Percutaneous Coronary Intervention. J Am Coll Cardiol 2013; 61: 2242-8
31. Huang MK, Hsu TF, Chiu YH*, et al.* Risk factors for acute kidney injury in the elderly undergoing contrast-enhanced computed tomography in the emergency department. J Chin Med Assoc 2013; 76: 271-6
32. Chen Y-L, Fu N-K, Xu J*, et al.* A Simple Preprocedural Score for Risk of Contrast-Induced Acute Kidney Injury After Percutaneous Coronary Intervention. Catheter Cardiovasc Interv 2014; 83: E8-16
33. Gao Y-m, Li D, Cheng H*, et al.* Derivation and validation of a risk score for contrast-induced nephropathy after cardiac catheterization in Chinese patients. Clin Exper Nephrol 2014; 18: 892-8
34. Tsai TT, Patel UD, Chang TI*, et al.* Validated contemporary risk model of acute kidney injury in patients undergoing percutaneous coronary interventions: insights from the National Cardiovascular Data Registry Cath-PCI Registry. J Am Heart Assoc 2014; 3: e001380
35. Tziakas D, Chalikias G, Stakos D*, et al.* Validation of a New Risk Score to Predict Contrast-Induced Nephropathy After Percutaneous Coronary Intervention. Am J Cardiol 2014; 113: 1487-93
36. Inohara T, Kohsaka S, Abe T*, et al.* Development and validation of a pre-percutaneous coronary intervention risk model of contrast-induced acute kidney injury with an integer scoring system. Am J Cardiol 2015; 115: 1636-42
37. Berisa F, Beaman M, Adu D*, et al.* Prognostic factors in acute renal failure following aortic aneurysm surgery. Q J Med 1990; 76: 689-98
38. Chen YC, Chen CY, Tien YC*, et al.* Organ system failures prediction model in intensive care patients with acute renal failure treated with dialysis. Ren Fail 2001; 23: 207-15
39. Fang JT, Tsai MH, Tian YC*, et al.* Outcome predictors and new score of critically ill cirrhotic patients with acute renal failure. Nephrol Dial Transplant 2008; 23: 1961-9
40. de Carvalho JR, Villela-Nogueira CA, Luiz RR*, et al.* Acute kidney injury network criteria as a predictor of hospital mortality in cirrhotic patients with ascites. J Clin Gastroenterol 2012; 46: e21-6
41. Pan H-C, Jenq C-C, Tsai M-H*, et al.* Risk Models and Scoring Systems for Predicting the Prognosis in Critically Ill Cirrhotic Patients with Acute Kidney Injury: A Prospective Validation Study. PLoS ONE 2012; 7: e51094
42. Schneider DF, Dobrowolsky A, Shakir IA*, et al.* Predicting acute kidney injury among burn patients in the 21st century: a classification and regression tree analysis. J Burn Care Res 2012; 33: 242-51
43. Chen CY, Lin YR, Zhao LL*, et al.* Clinical factors in predicting acute renal failure caused by rhabdomyolysis in the ED. Am J Emerg Med 2013; 31: 1062-6
44. Forni LG, Dawes T, Sinclair H*, et al.* Identifying the patient at risk of acute kidney injury: a predictive scoring system for the development of acute kidney injury in acute medical patients. Nephron Clin Prac 2013; 123: 143-50
45. McMahon GM, Zeng X, Waikar SS. A Risk Prediction Score for Kidney Failure or Mortality in Rhabdomyolysis. JAMA Intern Med 2013; 173:1821-8
46. Rodriguez E, Soler MJ, Rap O*, et al.* Risk Factors for Acute Kidney Injury in Severe Rhabdomyolysis. PLoS ONE 2013; 8 e82992
47. Slack AJ, McPhail MJ, Ostermann M*, et al.* Predicting the development of acute kidney injury in liver cirrhosis--an analysis of glomerular filtration rate, proteinuria and kidney injury biomarkers. Aliment Pharmacol Ther 2013; 37: 989-97
48. Slankamenac K, Beck-Schimmer B, Breitenstein S*, et al.* Novel Prediction Score Including Pre- and Intraoperative Parameters Best Predicts Acute Kidney Injury after Liver Surgery. World J Surg 2013; 37: 2618-28
49. Wang Y-N, Cheng H, Yue T*, et al.* Derivation and validation of a prediction score for acute kidney injury in patients hospitalized with acute heart failure in a Chinese cohort. Nephrol 2013; 18: 489-96
50. Kilic A, Grimm JC, Shah AS*, et al.* An easily calculable and highly predictive risk index for postoperative renal failure after heart transplantation. J Thorac Cardiovasc Surg 2014; 148: 1099-104
51. Kim JM, Jo YY, Na SW*, et al.* The predictors for continuous renal replacement therapy in liver transplant recipients. Transplant Proc 2014; 46:184-91
52. Simonini M, Lanzani C, Bignami E*, et al.* A new clinical multivariable model that predicts postoperative acute kidney injury: impact of endogenous ouabain. Nephrol Dial Transplant 2014; 29: 1696-701
53. Theocharidou E, Pieri G, Mohammad AO*, et al.* The Royal Free Hospital Score: A Calibrated Prognostic Model for Patients With Cirrhosis Admitted to Intensive Care Unit. Comparison With Current Models and CLIF-SOFA Score. Am J Gastroenterol 2014; 109: 554-62
54. Ahmed A, Vairavan S, Akhoundi A*, et al.* Development and validation of electronic surveillance tool for acute kidney injury: A retrospective analysis. J Crit Care 2015; 30: 988-93
55. Bell S, Dekker FW, Vadiveloo T*, et al.* Risk of postoperative acute kidney injury in patients undergoing orthopaedic surgery-development and validation of a risk score and effect of acute kidney injury on survival: observational cohort study. BMJ 2015;351: h5639
56. Dupont B, Delvincourt M, Kone M*, et al.* Retrospective evaluation of prognostic score performances in cirrhotic patients admitted to an intermediate care unit. Dig Liver Dis 2015; 47: 675-81
57. Grimm JC, Lui C, Kilic A*, et al.* A Risk Score to Predict Acute Renal Failure in Adult Patients After Lung Transplantation. Ann Thorac Surg 2015; 99: 251-7
58. Park MH, Shim HS, Kim WH*, et al.* Clinical Risk Scoring Models for Prediction of Acute Kidney Injury after Living Donor Liver Transplantation: A Retrospective Observational Study. PLoS ONE 2015; 10: e0136230
59. Gong Y, Xu H, Xu Z*, et al.* Comparison of prognostic value of two kinds of severity scoring systems for hospital mortality prediction of elderly patients with acute kidney injury. Aging Clin Exp Res 2012; 24: 74-8
